# Integrated Management of Childhood Illnesses implementation-related factors at 18 Colombian cities

**DOI:** 10.1186/s12889-020-09216-0

**Published:** 2020-07-16

**Authors:** Andrés Mauricio García Sierra, Jovana Alexandra Ocampo Cañas

**Affiliations:** Universidad de Los Andes, School of Medicine, Bogotá, Colombia

**Keywords:** Health care, Infant mortality, IMCI, Primary health care

## Abstract

**Background:**

Integrated Management of Childhood Illnesses (IMCI) is a strategy developed by the World Health Organization (WHO) and UNICEF in 1992. It was deployed as an integrated approach to improve children’s health in the world. This strategy is divided into three components: organizational, clinical, and communitarian. If the Integrated Management of Childhood Illnesses implementation-related factors in low- and middle-income countries are known, the likelihood of decreasing infant morbidity and mortality rates could be increased. This work aimed to identify, from the clinical component of the strategy, the implementation-related factors to Integrated Management of Childhood Illnesses at 18 Colombian cities.

**Methods:**

A quantitative cross-sectional study was performed with a secondary analysis of databases of a study conducted in Colombia by the Public Health group of Universidad de Los Andes in 2016. An Integrated Care Index was calculated as a dependent variable and descriptive bivariate and multivariate analyses to find the relationship between this index and the relevant variables from literature.

**Results:**

Information was obtained from 165 medical appointments made by nurses, general practitioners, and pediatricians. Health access is given mainly in the urban area, in the first level care and outpatient context. Essential medicines availability, necessary supplies, second-level care, medical appointment periods longer than 30 min, and care to the child under 30 months are often related to higher rates of Integrated Care Index.

**Conclusion:**

Health care provided to children under five remains incomplete because it does not present the basic minimums for the adequate IMCI’s implementation in the country. It is necessary to provide integrated care that provides medicine availability and essential supplies that reduce access barriers and improve the system’s fragmentation.

## Background

### IMCI strategy

In 1992, the World Health Organization (WHO) and UNICEF developed a strategy for children’s health care, known as Integrated care for childhood illnesses (IMCI). This strategy was designed as an integral approach to improve children’s health in the world [1]. The IMCI provides unified health care instead of separate management of diseases affecting children under five. Moreover, this approach also focuses on the reduction of morbidity and mortality rates associated with the most common diseases in childhood. The strategy is divided into three components: organizational, clinical, and community. The IMCI approach has introduced several ways to mitigate infant risks in several action fronts.

According to the WHO, about 68 million children around the world will die before reaching 5 years by 2030 [2]. Most of these deaths are caused by one of the following diseases or a combination of them: acute respiratory infection (ARI), acute diarrheal disease (ADD), measles, malaria, and malnutrition [1, 3]. 70% of such cases occur in low-income countries. Moreover, starting from the fact that the IMCI was created in line with the Millennium Development Goal (MDG) number four, which indicated the need to reduce by two-thirds the infant mortality by 2015, the correct application of the strategy would allow the reduction of the expected morbidity and mortality in the upcoming years [4].

On the other hand, and considering Alma-Ata’s statement, the conceptual framework of IMCI is close to Primary Health Care (PHC). Specifically, in several countries of the Americas region, where the strategy was introduced, there has been a “Primary Health Care for children” (PHC) [5]. Additionally, the IMCI focuses on the first contact of children with the health system, thus, it promotes health access and quality for this population group [6]. Similarly to the development of PHC, the IMCI has been deployed attending the specific needs and capacities of each country in the region [5].

### National coverage

Since 2004, more than 100 countries, including Colombia, have adopted the components of the IMCI. Specifically, the clinical component for the evaluation, treatment, and prevention of sick children, as well as, counseling to caregivers was mainly implemented [7]. When the strategy was introduced in Colombia this year, it was based on the right of every child to be treated with quality and warmth. It adopted a risk identification approach, of total integration, and was aimed at responding to the main causes of morbidity and mortality of children in the country for the next 10 years [7]. Despite all efforts of the Health Ministry to implement the strategy in all country’s departments, the main challenge was poor adherence by trained professionals in this strategy [6].

**Fig. 1:**
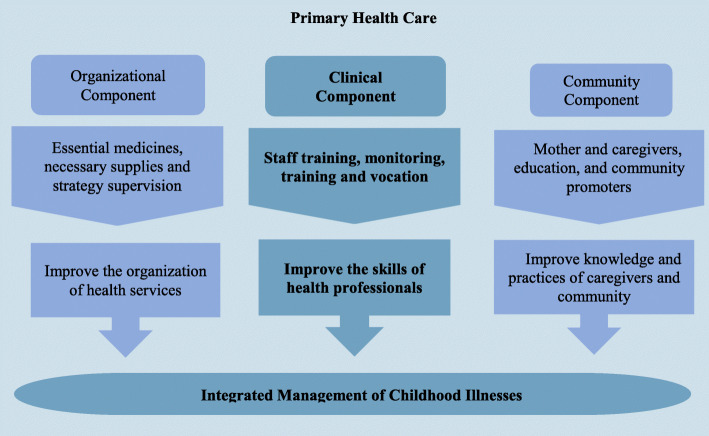
Conceptual framework of Integrated Management for Childhood Illnesses implementation-related factors. The figure was made by the authors regarding the key concepts found in the reference [8]

If the factors that influenced the implementation of the IMCI strategy had been considered in the country, it would have increased the probability of reducing the infant mortality rate from 19.5 deaths per 1000 live births in 1998 to 6.5 deaths in 2015. However, in 2013, there were 11.6 deaths per 1000 live births (6.7). Since its introduction in low-income countries, the IMCI strategy has shown positive results in reducing infant mortality. Unfortunately, factors such as the availability of medications, enough technical equipment, and permanent training for health professionals have not been studied in the Colombian context. Figure 1 illustrates the IMCI strategy implementation-related factors and benefits in the global context, regarding PHC [8].

### Implementation related factors

Kiplagat (2014) mentioned that in Tanzania factors such as personal training, monitoring, and vocation, lead to improve health care and improve adherence to treatments by families and infants [8]. It was also stated that the availability of medications, vaccines, the correct financing, and leadership by administrators can lead to the improvement of the quality of children’s care [8]. On the other hand, Rowe (2012) asserted that the presence of enough equipment, essential medicines, strategy supervision visits and number of trainings not only determine the performance of health workers, but also the level of coverage of the intervention [9]. Therefore, by gathering the basic minimums to offer a correct strategy, better health care for children under five could be achieved. Similarly, the strategy offers integrated management for childhood predominant illnesses, by achieving a reduction in infant mortality and morbidity [10]. Nevertheless, several international studies have shown that it is not carried out and it is presumed to be that the understanding of the motivations could improve health care [11].

Although the impact of the strategy has not been documented given the lack of national coverage [12], it has been identified a decrease in the infant mortality rate that seems to be related to the inclusion of the strategy in 2004 [13]. Moreover, there is no clarity about what factors determine the implementation of the IMCI in Colombia. In 2011, a document published by Universidad Nacional defined the care conditions provided from the clinical component of the strategy. Nevertheless, the implementation-related factors were not identified. Thus, in 2016, the Universidad de Los Andes in agreement with the Pan American Health Organization and the Health Ministry assessed the integrated health care received by children under five by practitioners formed in the clinical component of the strategy between 2012 and 2014.

The IMCI strategy provides children’s health monitoring through promotional, preventive, and therapeutic approaches, as well as interactions with the child, the family, health services, and other social sectors. Therefore, this work will show an integrated care index that involves general information, identification, evaluation, classification, treatment, and counseling to children and their caregivers in the most prevalent pathologies of childhood [14]. Moreover, framed in the clinical component of the strategy, this work is aimed at identifying the IMCI implementation-related factors at 18 Colombian cities.

## Methods

A cross-sectional study was performed with the database of the study conducted in Colombia by the Public Health group of Universidad de Los Andes in 2016 in agreement with the Pan American Health Organization and the Ministry of Health. The primary study was called “Comprehensive evaluation of children by the human talent trained in the clinical component of IMCI between 2,012 – 2,014”. Such work included the assessment of the consultation of 189 practitioners who were trained in the clinical component of the strategy between 2012 and 2014. The selection of these professionals was carried out through a probabilistic sampling with municipal and health care providers representativeness. Moreover, the primary study included the characterization of the consultation provided by professionals trained in IMCI that were working as health care personnel. This information was obtained directly from the PAHO Colombia website and the database was divided as shown in Table 1, where the components indicated each instrument with which the data were collected by the researchers of the primary study.

**Table 1:** Primary database information

Components	Description	Observations
A Component	Information about the health service and health personnel	75 evaluated institutions
B1 Component	Evaluation of the child from 2 months to 5 years	162 evaluated children
B2 Component	Evaluation of the child under 2 months	27 evaluated children
B3 Component	Applicability of IMCI and training course	189 evaluated professionals
C Component	Verification of support facilities	75 evaluated institutions
D Component	Final interview with mother or companion	189 interviews
F Component	Review of medical records	189 revised medical records

From the primary study, all the observations of the IMCI consultation (i.e., containing the total of the calculated indicators) were taken as the eligible population for this project. Subsequently, the indicators of all the components were joined, and the observations that contained missing data of the sociodemographic variables were dismissed (i.e., health service’s type, geographical area, complexity level, type of training, practitioner gender, type of practitioner, IMCI strategy supervision, health care provider, and consultation time).

To obtain all the available information, the observations containing the total data for the selected variables were included in this study. It was considered the calculation of the indicators for each component, as well as the observations that had a linking variable to make the information crossing. Repeated observations were found by the codes of the professionals observed and, therefore, 24 records were not used for the analysis. By having the complete information, a total of 165 effective observations, an integrated care index (ICI) was proposed, which correlated with each variable chosen. This index was taken as a continuous variable and was interpreted within the bivariate and multivariate analysis as the dependent variable. The structure of ICI was obtained from the WHO Health Facility Survey tool [15] and implies the following: For children under 2 months, 24 indicators (B2, C and D component) (Table 2) were obtained in the primary study, while children from 2 months to 5 years, 27 indicators were obtained in the primary study (B1, C, and D component) (Table 2). In this way, the ICI calculation for each observation involves adding the number of indicators that were obtained over the total of indicators for each age group. It should be noted that the indicators that were used to calculate this index were indicators designed and validated by PAHO Colombia.

**Table 2:** Indicators by each component from primary study

Component	Number of Indicators
B1 Component	21 indicators
B2 Component	18 indicators
C Component	5 indicators
D Component	1 indicator

With the total calculation of the ICI, a descriptive analysis was carried out through the selected variables and it was described concerning variables such as the type of service, the scope of the service, consultation time, and others. Consequently, the Student’s t-test and the z-test were used to assess the statistical significance of the difference in means and proportions, respectively, between the types of professionals in the variables considered. Subsequently, the relationship of the precoded variables with the ICI was estimated. Pearson correlation coefficients for continuous variables and Chi-square tests for categorical variables were used. Finally, a multivariate analysis was performed through a multiple regression by ordinary least squares with adjustment for robust standard errors. This analysis was aimed at analyzing the relationship between the ICI’s natural logarithm of each consultation with the independent variables that showed a relationship in the bivariate analysis, including the controls that the literature mentioned as relevant.

## Results

**Fig. 2:**
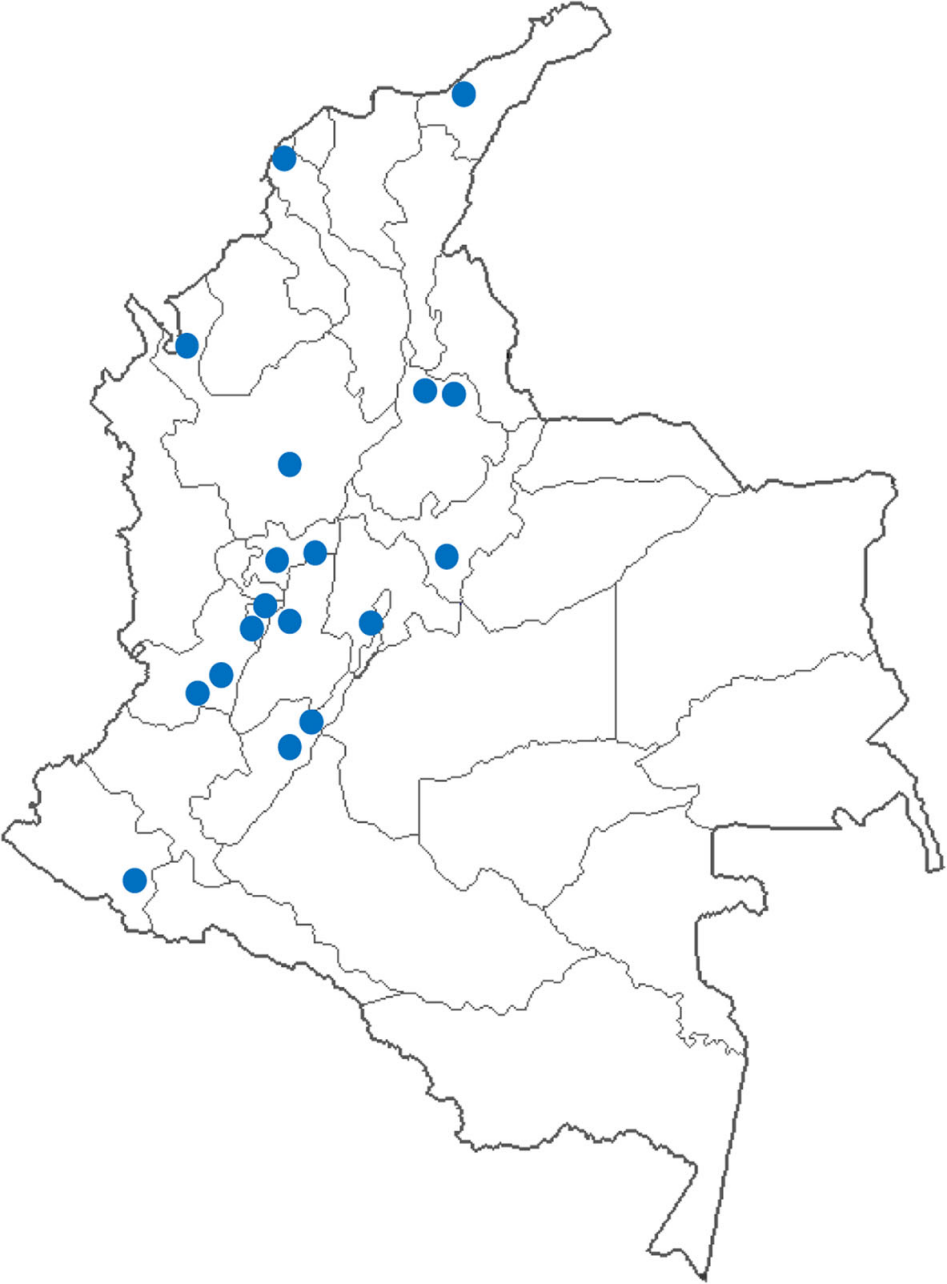
Distribution of research cities. The map was made by the authors using STATA 14 and the shapefile was obtained from the free open access ArcGIS website

**Fig. 3:**
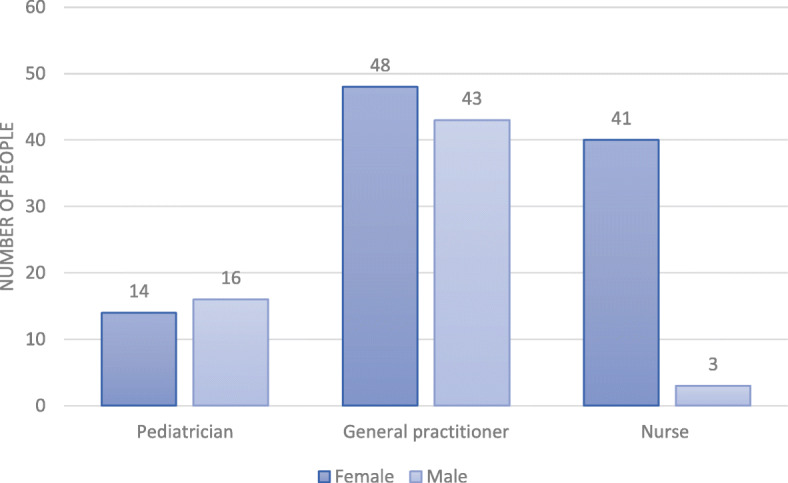
Distribution of health professionals by gender. Light blue shows the number of men and dark blue the number of women

The evaluation was obtained from 165 consultations carried out with children under five. These evaluations were distributed at 18 municipalities and 12 departments, including Bogotá as a department. The municipalities included cities such as Medellín, Cartagena, Cali, Neiva, Bucaramanga, among others (Fig. 2). Likewise, the evaluation was obtained for the 165 health professionals distributed at 70 health care providers. These institutions represented the majority of the centers with the highest influx of patients in the municipalities evaluated, as well as the provision of services by insurers from both the contributory and subsidized systems. The health professionals evaluated included 103 women and 62 men. A total of 30 pediatricians, 91 general practitioners, and 44 nurses distributed by sex were evaluated, as shown in Fig. 3.

Table 3 shows the distribution of the evaluated practitioners by the variables of interest and discriminated by type of practitioner, including the professional type mean for the continuous variables. The mean age for all types of professionals was 40 years, the mean age of children was 20 months, the meantime of each consultation was 31.5 min, and the professionals had trained at least twice in the strategy. There were no statistically significant mean differences in these variables between physicians and nurses (*P* > 0.05). The type of training that predominated was virtual, however, the difference was not statistically significant (P > 0.05). The most trained professionals were the pediatricians, however, the ones who most attended the children were the general practitioners, as evidenced in Fig. 3. Similarly, at least 33 health professionals were trained less than three times in the strategy. This relationship was less clear in the group of general practitioners.

**Table 3:** Sample Characterization

	Pediatrician (*n* = 30)	General Practitioner (*n* = 91)	Nurse (*n* = 44)
Practitioner’s gender			
Male	16	43	3
Female	14	48	41
Practitioner’s age^ab^	49.29 (11.60)	39.57 (9.69)	33.19 (8.20)
Medical consultation’s time^ac^	30.46 (11.53)	32.56 (24.76)	34.34 (12.67)
Child’s age ^ad^	15.13 (17.40)	22.27 (17.72)	21.51 (17.57)
Child’s gender			
Male	16	45	26
Female	14	46	18
IMCI Chart booklet			
Yes	18	31	19
No	12	60	25
IMCI Medical records			
Yes	18	56	29
No	12	35	15
Supplies			
Yes	11	55	20
No	19	36	24
Complexity level			
First level	14	71	34
Second level	7	15	9
Third level	9	5	1
Geographical area			
Rural	0	4	3
Urban	30	87	41
Health attention type			
Public	3	58	29
Private	27	33	15
Number of trainings^a^	3.30 (2.11)	2.04 (1.01)	2.09 (1.58)
Training type			
Virtual	14	40	21
Presential	11	38	17
Mixed	5	13	6
Strategy supervision			
Yes	11	35	15
No	19	56	29
Oral rehydration unit			
Yes	11	16	7
No	19	75	37
Vaccine application centre			
Yes	2	16	4
No	28	75	40
Essential medicines			
Yes	6	3	1
No	24	88	43
Health services type			
Scheduled consultation	23	76	42
Urgencies	7	15	2

^a^ Mean (SD)

^b^ Age in years

^c^ Time in minutes

^d^ age in months

**Fig. 4:**
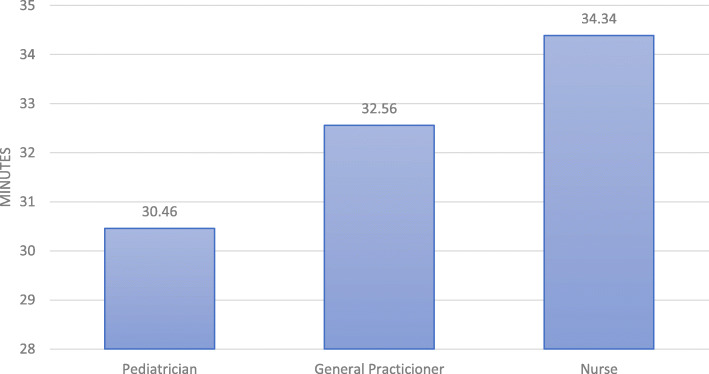
Average length in minutes of consultation by Health practitioners

Figure 4 shows the consultation time distribution spent by the health practitioners. The average time for practitioners was 31 min per consultation. On average, the nurses spent more time doing the IMCI consultation, but this difference was not statistically significant (*P* > 0.05). Of the 165 evaluated consultations, 119 corresponded to a first level of care, 31 to a second level, and 15 to a third level. Similarly, 158 professionals were assessed in the urban area, while 7 were from rural areas, 90 consultations were made in public health services, while 75 were in private services.

Regarding ICI distribution from the 165 health professionals, the minimum value of the index was 0 while the highest was 0.62, for a range from 0 to 1. The above indicates that the model may explain the correlation between the variables only in this range. The average value of the index was 0.36 for all the consultations without differences by type of professional (*p* < 0.05). With the achievement of the distribution of the ICI, the correlation coefficients shown in Table 4 were obtained. The variables that showed a positive association with the ICI and that had statistical significance were essential medicines, scheduled consultation, virtual training, IMCI evaluation method, the second level of complexity, necessary supplies, IMCI medical records, IMCI chart booklet and consultation time. That is, the professionals who had mixed training and a method to evaluate the IMCI, who attended at a second level, who had the necessary supplies, who had the IMCI medical records with the IMCI chart booklet and who invested more time in their consultations, obtained a higher ICI. The variables that showed a negative association with the ICI and that had statistical significance were the geographic scope and the age of the child. That is to say, the professionals who attended in an urban environment and who attended larger children had lower integrated attention rates.

**Table 4:** Correlation coefficients for the selected variables

	ICI®	*P*-Value	CI 95%
Women practitioner	−0.02	0.37	−0.06 0.02
Schedule consultation	0.07	0.00	0.03 0.11
Essential medicine	0.09	0.05	0.00 0.18
Unsupervised strategy	−0.04	0.08	− 0.08 0.00
Blended training	0.06	0.04	0.00 0.12
Number of trainings	0.01	0.23	−0.01 0.02
IMCI assessment	0.07	0.00	0.03 0.11
Private health care provider	0.01	0.77	−0.04 0.05
Urban area	−0.11	0.03	−0.21 -0.01
Second level complexity	0.10	0.00	0.05 0.15
Necessary supplies	0.14	0.00	0.10 0.17
IMCI medical records	0.07	0.00	0.03 0.11
IMCI chart booklet	0.11	0.00	0.07 0.15
Child age	−0.00	0.01	−0.00 -0.00
Consultation time	0.00	0.00	0.00 0.01
Nurse	−0.03	0.29	−0.10 0.03
Practitioner age	0.00	0.35	−0.00 0.00

The results from the multiple regression model are shown in Table 5. The explanatory variables included in this model provide a 51% explanation of the dependent variable. The statistically significant variables were essential medicines, IMCI assessment, the second level of complexity, necessary supplies, child age, and consultation time (*p* < 0.05). In this sense, it was obtained that the provision of essential medicines where the service is provided, increased the integrated care rate by 37%. Moreover, when children under five were attended at a second level of complexity, with the necessary supplies and with the provision of an IMCI assessment method, the ICI was improved by 18, 34, and 15%, respectively. On the other hand, it was found that consultations that took less than 30 min reflected a decrease in the ICI of more than 10%. Finally, in the consultations where children between 40 and 60 months of age were treated, an average reduction of 30% was obtained in the ICI compared to those under 30 months. Table 6 shows the percentage changes from the multiple regression model.

**Table 5:** Multiple regression model with adjustment for robust standard errors

	ICI®	P-Value	CI 95%
Essential medicines	0.09	0.02	0.01 0.17
Scheduled consultation	− 0.04	0.12	− 0.09 0.01
Virtual training	− 0.01	0.77	− 0.04 0.03
Blended training	0.00	0.85	−0.05 0.06
IMCI assessment	0.07	0.00	0.04 0.11
Urban area	−0.05	0.03	−0.13 − 0.03
Second level complexity	0.05	0.02	0.01 0.10
Third level complexity	0.04	0.24	−0.03 0.10
Necessary supplies	0.10	0.00	0.06 0.14
Not IMCI chart booklet	-0.03	0.14	−0.06 0.01
Not IMCI medical records	−0.02	0.22	−0.06 0.01
Child age	−0.00	0.04	−0.00 -0.00
Consultation time	0.00	0.01	0.00 0.00

**Table 6:** Multiple regression model with robust standard error adjustment for percentage changes

	ln ICI®	RSE
Essential medicines**	0.37	0.15
Scheduled consultation	−0.15	0.12
Virtual training	0.03	0.08
Blended training	0.01	0.09
IMCI assessment**	0.15	0.07
Urban area	0.09	0.10
Second level complexity**	0.18	0.07
Third level complexity	0.03	0.19
Necessary supplies***	0.34	0.06
IMCI chart booklet	0.05	0.08
IMCI medical records	0.10	0.07
10 min consultation*	−0.23	0.13
20 min consultation**	−0.16	0.08
40 min consultation	0.02	0.09
50 min consultation	−0.01	0.12
60 min consultation	0.00	0.10
70 min consultation	0.14	0.11
1–12 months child	−0.15	0.10
1-year child	−0.11	0.11
2-year child	−0.04	0.11
4-year child**	−0.22	0.11
5-year child***	−0.38	0.13

*** *p* < 0.01, ** *p* < 0.05, * *p* < 0.10

## Discussion

### IMCI strategy

IMCI is a cost-effective strategy that improves the quality of medical care for the most common causes of morbidity and mortality in children under five through evidence-based medicine [16]. However, this study showed low rates of integrated care management, reflecting the lack of monitoring of the strategy in strict control by the regulatory agencies. Similarly, in countries of the region such as Peru or Bolivia, it has not been adequately documented owing to low national coverage [12, 17]. This similarity may be due to the lack of interest in the implementation of the strategy by health professionals and health institutions for the proper approach of children, as mentioned in the literature.

### National Coverage

A systematic literature review carried out by Amaral and Victoria in 2008, concluded that the training improves the evaluation, communication, and the rational use of antibiotics. In Colombia, it is not known whether continuing medical education is improving skills in other areas, such as vaccines and nutritional counseling, among others, as well as the magnitude of these benefits [18]. The results of this study indicate that the number of training sessions does not affect the quality of care for children under five. However, professionals who received mixed training did show better results without statistical significance. This may suggest that this type of training should be received by health practitioners to improve IMCI.

### Implementation related factors

Rowe in 2012 mentioned that the presence of sufficient equipment, essential medicines, strategy supervision’s visits, and the number of trainings, not only determine the performance of health workers, but also the level of intervention coverage [9]. Accordingly, the availability of the necessary supplies and essential drugs could be influential factors in improving the applicability of IMCI. Strengthening these factors would result in an enhancement of the strategy implementation process. Furthermore, a systematic review by Goga et al. explored the adequacy of implementation as a confounding factor in clinical performance, although within a slightly different context, decreased training time would seem to have the same effect [19]. For this study, consultation time and supervision of the strategy by control entities showed an association with better medical care. The correct allocation of time in consultation and receive the support of the institutions where IMCI is used, will allow a strengthening of the implementation of the strategy. In the first analyzes shown, the evaluation, classification and treatment tables, the related training materials (i.e., known as the IMCI chart booklet and IMCI handbook), the training materials (i.e., to improve communication with parents during the evaluation of their children) and the follow-up guides to health personnel (i.e., to support the effective application of the clinical component of the IMCI strategy) [4], have a positive association with the quality of care, which is aligned with the fundamental principles of the IMCI strategy. However, this association loses its significance when included in the multivariate model. Despite the above, it is necessary to continue promoting the use of these tools since it allows professionals to order the consultation and obtain better clinical results.

Concerning the health care context, it was found that children who were treated in urban facilities received a lower level of care compared to the rural area, indicating the lack of availability in the urban area. The mean time for consultations in rural areas lasts longer than those in the urban environment. Considering that time plays an important role in integrated care, this finding may be influenced by the relationship discussed above and may offer future research development topics to know the optimal consultation time for IMCI. Besides, it was also found that the second level of complexity provides a positive association with the integrated care index. The fact that younger children receive more attention and require more specialized services would be an explanation of this finding. Although the IMCI strategy is thought to be the first contact of the population with the health system, the previously discussed finding indicates that it can be extrapolated to the other levels of care, and the attention in the first levels must be reinforced.

Although associations were found between other factors that influence integrated attention to childhood diseases, when possible relationships were included in a multivariate model, they lost their statistical significance. Aspects such as having an IMCI history, having a chart of procedures, having types of training that motivate the health professionals, and conducting the consultation in the context of the external consultation should not be overlooked. These factors could contribute to an alternate way to achieve better care for children under five.

### Limitations

Being a cross-sectional study, the greatest limitation is the impossibility of finding causal relationships. However, this study tried to adjust the homoscedasticity principle when adjusting the multivariate model with robust standard errors. Likewise, it is important to consider that the results consist only of internal validity, in such a way that they cannot be applied outside the observations of the primary database. Despite the above, the results obtained constitute a starting point for further studies throughout Colombia.

Moreover, the primary database had some level of bias because this ended up being a convenience sampling. This implies that the results will only be inferred to the health care providers at the time of the data collection, without a national representativeness. Finally, this work does not provide any insight or explanation of the results from the different actors involved in the process of care to children. Thus, it is not possible to characterize the dynamics that are immersed in the implementation of the clinical component of the IMCI strategy.

## Conclusions

The health care provided to children under five remains incomplete due to the lack of basic minimums for the correct IMCI implementation in Colombia. It is required an integrated care that provides medicines and necessary supplies, reducing access barriers and improving the fragmentation of the system.

Moreover, the fracture of the system is evidenced in the achievement of integrated care in the second level of care and rural areas. An articulation between the clinical, institutional, and community component is required, allowing the improvement in the factors.

Despite the evidence found, it is important to carry out studies with another methodological design and with another type of sampling to establish causality and national representativeness, respectively.

The general benefit of the IMCI strategy is to contribute to the reduction of the under-five mortality rate, which is in line with MDG 4. The identification of the factors that influenced integrated care for prevalent diseases of childhood will help government agencies and decision-makers to improve their implementation in Colombia. These findings and recommendations will be available to the territorial entities and results will also be shared with other stakeholders that may include: service providers, health professionals, and universities.

## Data Availability

The databases from which the present study is derived can be found on the PAHO Colombia website. They are freely accessible and have an assignment code for each observation to keep the information confidential.

## Abbreviations

ADD: Acute Diarrheal Disease
ARI: Acute Respiratory Infection
Complexity level: The level of complexity of a health care provider is determined by the number of differentiated tasks that make up the overall activity of an establishment and the degree of development achieved by them. The first levels of care give basic medical services while the third levels give more specialized services
ICI: Integrated Care Index
IMCI: Integrated Management for Childhood Illnesses
IMCI Medical Records: Availability of IMCI medical records at health care providers
IMCI presential training: Face-to-face trainings for professionals who work in health institutions, community health programs, and other childhood care programs to contribute to providing integrated management to childhood illnesses
IMCI scheduled consultation: Is the consultation given with an integrated approach to child health that focuses on the general well-being of the child. Preventive and curative components are covered for its application and it is offered as scheduled consultations
IMCI urgency consultation: Is the consultation given with an integrated approach to child health that focuses on the general well-being of the child. Therapeutic and curative components are covered for its application and it is offered as consultations at ER
IMCI virtual training: Training oriented through a virtual platform where the practices were based on the simulation of cases
MDG: Millennium Development Goal
PAHO: Pan American Health Organization
PHC: Primary Health Care
Strategy supervision: Health care provider accompaniment to each consultation offered under the IMCI strategy that allows identifying opportunities for improvement and thus offering better health care
UNICEF: United Nations Children’s Fund
WHO: World Health Organization
